# Rise of *Ruppia* in Chesapeake Bay: Climate change–driven turnover of foundation species creates new threats and management opportunities

**DOI:** 10.1073/pnas.2220678120

**Published:** 2023-05-30

**Authors:** Marc J. S. Hensel, Christopher J. Patrick, Robert J. Orth, David J. Wilcox, William C. Dennison, Cassie Gurbisz, Michael P. Hannam, J. Brooke Landry, Kenneth A. Moore, Rebecca R. Murphy, Jeremy M. Testa, Donald E. Weller, Jonathan S. Lefcheck

**Affiliations:** ^a^Department of Biological Sciences, Virginia Institute of Marine Sciences, College of William and Mary, Gloucester Point, VA 23062; ^b^University of Maryland Center for Environmental Science, Cambridge, MD 21613; ^c^Environmental Studies Program, St Mary’s College of Maryland, St Mary's City, MD 20686; ^d^National Park Service, Southwest Alaska Inventory and Monitoring Network, Anchorage, AK 99501; ^e^Maryland Department of Natural Resources, Annapolis, MD 21401; ^f^Chesapeake Bay Program Office, University of Maryland Center for Environmental Science, Annapolis, MD 21401; ^g^Chesapeake Biological Laboratory, University of Maryland Center for Environmental Science, Solomans, MD 20688; ^h^Smithsonian Environmental Research Center, Edgewater, MD 21037; ^i^Tennenbaum Marine Observatories Network, MarineGEO, Smithsonian Environmental Research Center, Edgewater, MD 21037

**Keywords:** phase shift, pollution, resilience, resistance, seagrass

## Abstract

Global change has converted many structurally complex and ecologically and economically valuable coastlines to bare substrate. In the structural habitats that remain, climate-tolerant and opportunistic species are increasing in response to environmental extremes and variability. The shifting of dominant foundation species identity with climate change poses a unique conservation challenge because species vary in their responses to environmental stressors and to management. Here, we combine 35 y of watershed modeling and biogeochemical water quality data with species comprehensive aerial surveys to describe causes and consequences of turnover in seagrass foundation species across 26,000 ha of habitat in the Chesapeake Bay. Repeated marine heatwaves have caused 54% retraction of the formerly dominant eelgrass (*Zostera marina*) since 1991, allowing 171% expansion of the temperature-tolerant widgeongrass (*Ruppia maritima*) that has likewise benefited from large-scale nutrient reductions. However, this phase shift in dominant seagrass identity now presents two significant shifts for management: Widgeongrass meadows are not only responsible for rapid, extensive recoveries but also for the largest crashes over the last four decades; and, while adapted to high temperatures, are much more susceptible than eelgrass to nutrient pulses driven by springtime runoff. Thus, by selecting for rapid post-disturbance recolonization but low resistance to punctuated freshwater flow disturbance, climate change could threaten the Chesapeake Bay seagrass’ ability to provide consistent fishery habitat and sustain functioning over time. We demonstrate that understanding the dynamics of the next generation of foundation species is a critical management priority, because shifts from relatively stable habitat to high interannual variability can have far-reaching consequences across marine and terrestrial ecosystems.

## Significance

Climate change creates ecosystems with fundamentally different conditions and species than in the past. In the Chesapeake Bay, rising temperatures have interacted with nutrient pollution to halve the cover of the historically abundant but climate change-vulnerable seagrass, eelgrass. In contrast, the heat-tolerant and opportunistic seagrass, widgeongrass, has nearly doubled in extent. Widgeongrass’ rapid recolonization ability has fueled the two largest recorded increases in the Chesapeake Bay seagrasses since 1984, but also a devastating die-back in 2019. Shifting species dominance has created an ecosystem that is stochastic but also is influenced more by regional nutrient management than global climate change, leading to new opportunities and challenges to maintain healthy and viable coastlines.

Climate change and human activities are drastically altering the structure and function of ecosystems worldwide, particularly along the coasts that integrate across both land- and ocean-based stressors (1–4). These stressors have fragmented or eliminated structured coastal habitat around the world (5–8), yet in many cases, climate change is simultaneously promoting the turnover of dominant foundation species. For example, higher winter temperatures have allowed mangroves to extend their range into areas historically dominated by salt marshes and oyster reefs in five continents (9–11); warmer winters and summers have facilitated invasive pacific oysters replacing native blue mussels in the Wadden Sea (12); and overfishing, heatwaves, and nutrient pollution have promoted macroalgae dominance over hard corals throughout the Caribbean and Indo-Pacific (13). These climate-induced shifts appear to be particularly prevalent for extensive seagrass ecosystems in the Mediterranean, Caribbean, Atlantic, and Pacific. In these systems, species-specific differences in heatwave tolerance and recovery have changed dominant species identity (14), and rising temperatures are pushing invasive (sub)tropical species into temperate meadows (15), and may even expand temperate species into polar habitats (16). While global change may select for climate-tolerant foundation species that employ fast-growing, opportunistic, and/or invasive life history strategies, shifting species dominance can generate different ecosystem dynamics and compromise the consistent delivery of historically valued services (17–20). Predicting the future composition of coastal ecosystems poses a significant challenge to resource managers seeking to preserve valuable habitats in a rapidly changing world.

**Fig. 1.**
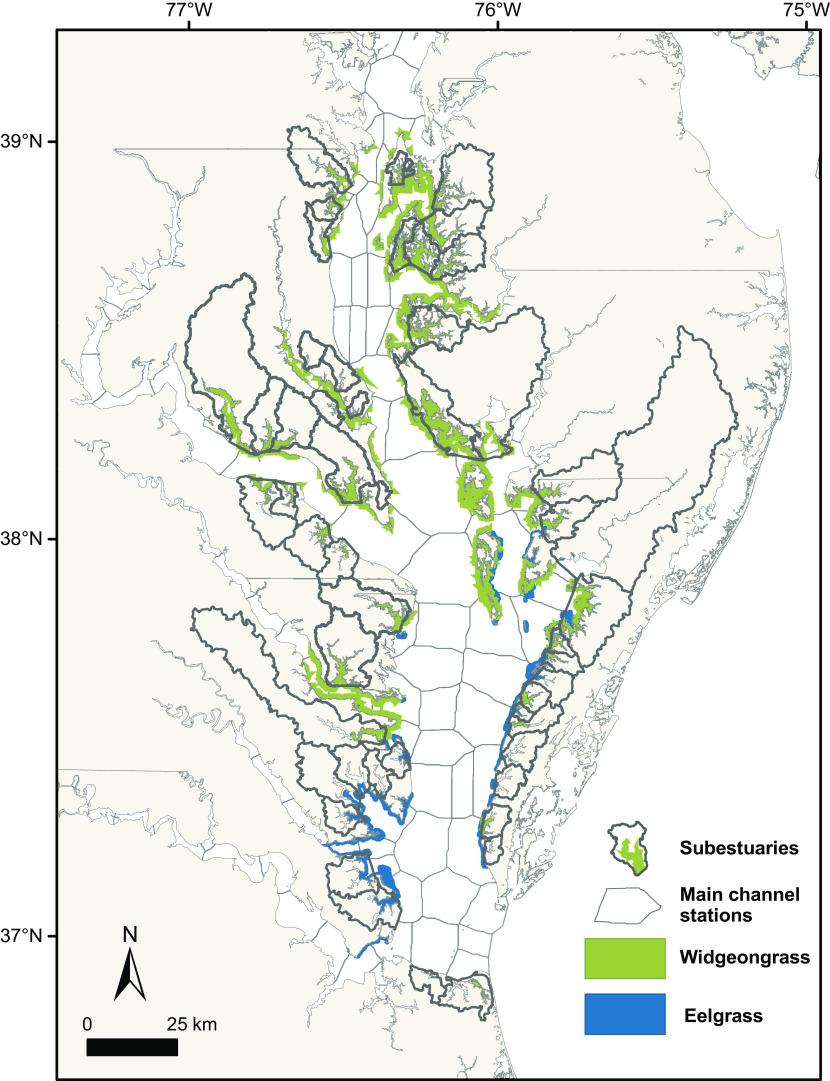
Widgeongrass (green, *R. maritima)* and eelgrass (blue, *Z. marina*) seagrass meadow coverage in the subestuaries (thicker polygons, subestuary analysis) and main channel sites (thin polygons, main channel analysis) of Chesapeake Bay in 2019. Eelgrass currently dominates the lower Chesapeake Bay but occupied both that area and current-widgeongrass dominated area until the early 1970s. Widgeongrass has been observed in varying densities throughout these areas from 1984 to 2019 but now forms dominant monocultures in the green area. To aid visualization, meadow coverage depicted is the maximum cover observed at any time in our survey.

The Chesapeake Bay, USA, is one of the largest and most-populated estuarine systems in the world and has a long history of documented changes in foundation species along its shorelines, such as the near extirpation of oysters in the 20th century (21). Of the ~23 species of aquatic marine, brackish, and freshwater plants that provide key habitat in the Chesapeake Bay (22), two seagrasses, eelgrass (*Zostera marina*) and widgeongrass (*Ruppia maritima*), occur from the Atlantic up through the middle of the basin (Fig. 1), while the remaining species occupy lower salinity areas closer to freshwater sources in the upper reaches of the bay (23). For much of the 20th century, the majority of the Chesapeake Bay was dominated by eelgrass (23, 24), but decades of water quality and habitat degradation from a rapidly growing human population, in conjunction with Tropical Storm Agnes of 1972, led to widespread eelgrass decline in the early 1970s and retreat to the southern waters (25). The next two decades saw a trend of recovery, which was later reversed due to heat stress induced by increasing mean summer temperatures and heatwave intensity and frequency: These climate change-driven stressors have reduced total eelgrass cover by 57% since its post-Agnes peak in the early 1990s (26).

The other seagrass historically prevalent in the lower estuary, the cosmopolitan species widgeongrass, has been projected to replace eelgrass in many areas due to its high-temperature tolerance, a long-lasting seed bank, and a large source population (23, 27, 28). Yet the consequences of widgeongrass replacing eelgrass are poorly understood for two major reasons. First, widgeongrass meadows have undergone dramatic fluctuations in area that far exceed anything observed for eelgrass [e.g., reported 70 to 80% meadow loss in 1 to 3 y, 600 to 900% meadow recovery over 3 y (29)]. Second, their shorter, thinner blades and shallower root depths provide different habitat structure that may alter the flow of nearshore goods and services (30, 31). These differences challenge both scientists and managers seeking to restore the Chesapeake Bay ecosystems and enhance human well-being. To bolster future estuarine response to climate change, we must leverage long-term data sources to quantify the current extent of this shift in species dominance and describe how human and climate controls on widgeongrass abundance may differ from the species it has replaced across management-relevant spatiotemporal scales.

Here, we use a 36-y record of aerial monitoring and ground surveys to quantify the recent shift in the identity of the dominant seagrass across the Chesapeake Bay. Then, we link this shift to known stressors including climate change, watershed nutrient- and sediment-loading, and water column biogeochemistry in two spatially explicit analyses covering 26,000 ha of shallow waters (Fig. 1). We first quantify the effect of land-use and local watershed nutrient and sediment inputs, including from both nonpoint sources (e.g., land runoff) and point sources (e.g., wastewater treatment facilities), on annual seagrass areal cover in 35 independent widgeongrass-dominated embayments (i.e., “subestuaries”). Our subsequent analysis describes the effect of abiotic variables in the water column (e.g., total nitrogen and phosphorus, salinity, water clarity) on annual seagrass areal cover adjacent to 51 permanent environmental monitoring stations in widgeongrass-dominated major rivers and tributaries (i.e., “main channel”). We build on recent work examining general trends and drivers of all species of submersed aquatic vegetation (i.e., “SAV”) in aggregate throughout the Chesapeake Bay (32) with new species-specific analyses and up-to-date data covering both a record-setting recovery and the most dramatic vegetative cover loss observed since 1973. We highlight emerging climate threats for widgeongrass, demonstrate the applications of our analysis for re-evaluating seagrass and coastal management goals as dominant species identity changes, and outline the functional implications of opportunistic, climate-tolerant coastal foundation species dominance in the future. We seek to inform short- and long-term conservation strategies for shifting foundation species across coastal ecosystems, especially as climate change continues to simultaneously alter environments and drive species turnover.

## Results and Discussion

**Fig. 2.**
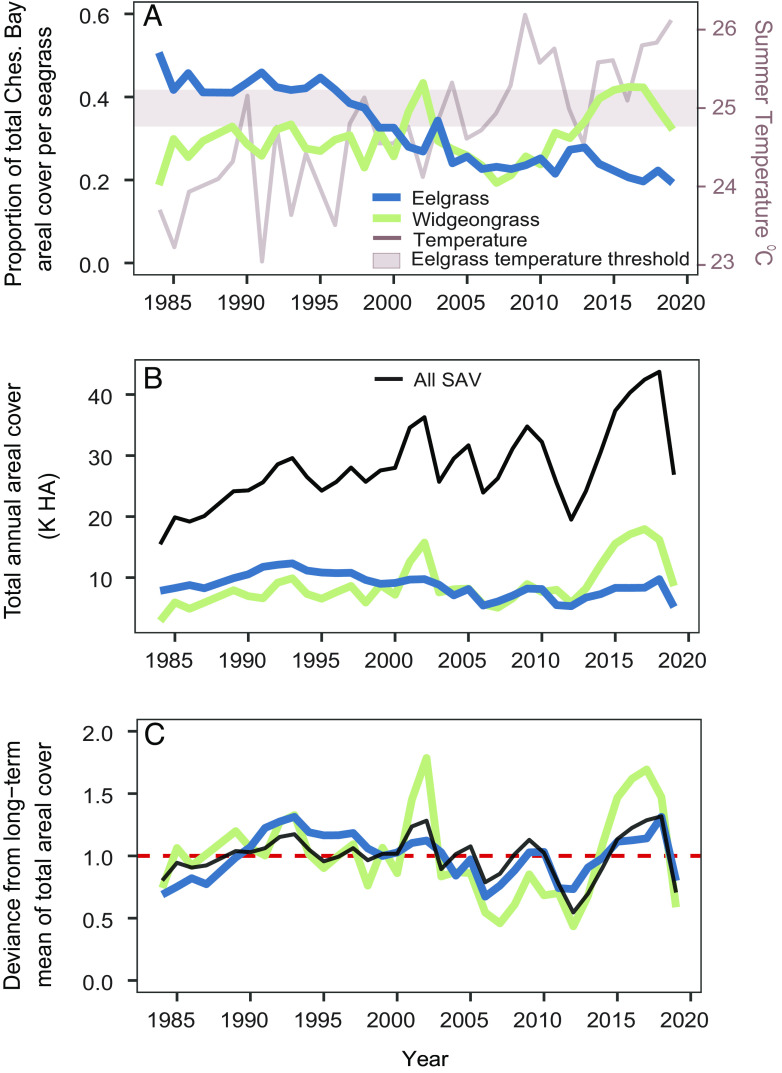
(*A*) Shifting spatial dominance over time of the two major seagrass foundation species in the lower Chesapeake Bay as mean summer temperatures (red line) increase. Proportion of total Chesapeake Bay SAV covered by the previously dominant eelgrass (blue) has declined while the proportional area covered by widgeongrass (green) has increased. Eelgrass begins to experience heat induced die-off at ~25 °C mean summer temperature (red shaded horizontal line), while widgeongrass temperature maxima are not reached in Chesapeake Bay. (*B*) Total areal cover of all Chesapeake Bay SAV coverage (total ha of all 23 species of seagrasses and aquatic plants, black) fluctuates with the largest gains and die-back of widgeongrass (green) over the last 20 y, while changes in eelgrass (blue) are of much lower magnitude. (*C*) Deviance around the long-term Chesapeake Bay mean areal cover (ha) for widgeongrass (green) and eelgrass (blue) indicate much larger year to year variability in widgeongrass cover than eelgrass cover. Points above or below the dashed red line at 1.0 depict years where mean cover was higher or lower than the average cover from 1984-2019. Total Chesapeake Bay SAV areal cover (black) has also been more variable since widgeongrass became the dominant space-holding foundation species after 2000.

From the beginning of our annual aerial surveys in 1984 until the early 2000s, eelgrass was the dominant foundation species in the Chesapeake Bay, making up over 40% of the total SAV areal coverage until the late 1990s (Fig. 2*A*). However, increasing summertime temperatures and lower water clarity have reduced eelgrass to just 19% of all SAV across the estuary in 2019 (Fig. 2*A*). In contrast, widgeongrass expanded rapidly during this time and has comprised as much as 43% of all Chesapeake Bay SAV area (i.e., in 2003 and 2015 to 2017). Accordingly, this new era of widgeongrass dominance corresponds with the two most significant peaks in Bay-wide SAV, 2001 to 2002 and 2013 to 2018. During the second peak, widgeongrass more than doubled its total area from 2013 to 2018 to fuel the recent record-setting levels of Bay-wide SAV (43,736 ha in 2018, 287.7% increase since 1984; Fig. 2*B*). However, focusing on these recent peaks ignores some troubling patterns: widgeongrass loss following these gains was also responsible for the largest recorded die-backs in the Chesapeake Bay history: −10,575 ha in 2003 and −16,871 ha in 2019. In the latter case, widgeongrass area halved over the course of just one year. In fact, total SAV cover has become increasingly variable in the 20 y since widgeongrass became dominant. In 50% of these recent years (i.e., 1999 to 2019), the Chesapeake Bay has either gained or lost at least 20% total SAV area from the previous year, and widgeongrass cover has been ~3× more variable than historical eelgrass cover (Fig. 2*C*; D = 0.34, *P* = 0.032). These patterns prompt questions regarding how these two species compare in their responses to the different types of year-to-year changes in the environment.

**Fig. 3.**
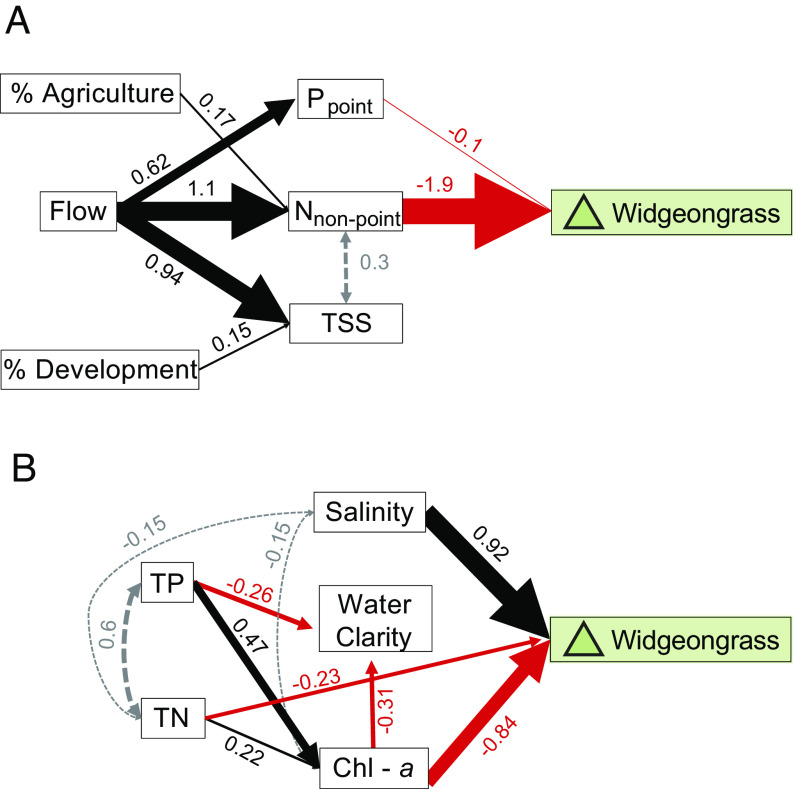
(*A*) Subestuary structural equation model (Fisher’s C = 6.505, *P* value = 0.771, df = 10), depicting the effect of watershed and nutrient inputs on widgeongrass change in subestuaries and embayments, and (*B*) main channel structural equation model (Fisher’s C = 1.71, *P* value = 0.789, df = 4), depicting the effect of springtime water column biogeochemistry on widgeongrass change in major rivers and tributaries of Chesapeake Bay. Arrow width is proportional to the standardized effect size given next to the black (positive effect), red (negative effect), and grey dotted (correlation effect) arrows. Nonsignificant relationships (*P* > 0.05) have been omitted for clarity. See *SI Appendix* for more model details.

To describe the anthropogenic and climate drivers of annual change in widgeongrass meadows, we use structural equation modeling to build (1) a single causal network of watershed loading controls across ~8,000 ha within 35 subestuaries, a representative suite of small independent embayments more strongly controlled by their local watersheds than the major baywide drainage basin; and (2) a single causal network of seasonal water quality controls across ~18,000 ha of widgeongrass habitat distributed among 51 water quality monitoring stations throughout the major rivers and channels of the Chesapeake Bay. Together, we find that annual widgeongrass cover (ha/year) is most negatively affected by increased watershed nutrient pollution (Fig. 3*A*), and most positively correlated with high springtime salinities in the water column (Fig. 3*B*). These analyses confirm that widgeongrass responds to distinctly different controls than the known negative drivers of eelgrass in the Chesapeake Bay, namely high temperature alone or simultaneous poor water clarity and high-temperature events (26). Additionally, while neither watershed nutrients nor interannual variation in salinity is broadly linked to eelgrass dynamics in the Chesapeake Bay (26, 28), we identified high salinity years, a proxy for low inflows, as the most influential indicator of annual widgeongrass areal expansion. Our two analyses together establish that temporal variation in the large-scale processes that influence local water quality conditions drive alternating boom–bust cycles in widgeongrass monocultures.

**Fig. 4.**
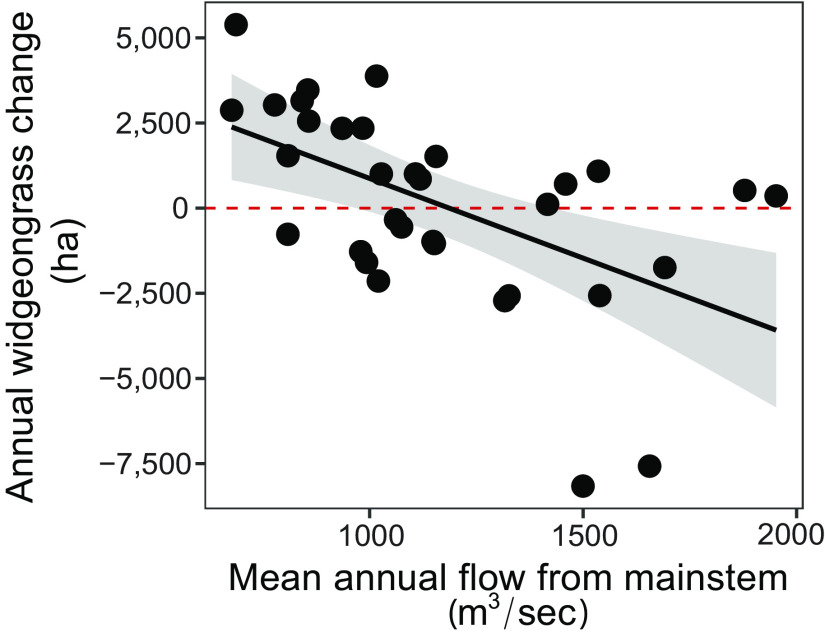
Annual discharge (mean cubic meters /second) from the major freshwater source into the main channel Chesapeake Bay, the Susquehanna River, is negatively correlated with total widgeongrass change in areal cover (R^2^ = 0.411).

High springtime runoff (i.e., flow from both elevated precipitation and riverine discharge) carries fertilizer, sediment, and other nonpoint sources of nutrients off the watershed (Fig. 3*A*). Subsequently greater springtime nutrient concentrations in the water column stimulate phytoplankton blooms (i.e., high chlorophyll-*a* in our model) and overgrowth by epiphytic algae directly on the seagrass blades, reducing light penetration and leading to significant widgeongrass retreat in wet years (Fig. 3*B*) (32–34). Because neither chl-*a* nor light availability alone cause eelgrass die-off and because both species can be vulnerable to epiphytic algal overgrowth, we can point to widgeongrass’ greater responsiveness to flow-driven nutrient pollution as the cause of shifting large-scale seagrass dynamics in the Chesapeake Bay. Now, rapid meadow recovery can exceed previous peaks under low flow conditions (Fig. 2*B*). In fact, annual run-off rates now may be an important marker of large-scale seagrass recolonization as the lowest annual river flow rates out of the headwater of the Chesapeake Bay, the Susquehanna River, correlate with the largest annual gains in total widgeongrass (Fig. 4; R^2^ = 0.41, F_1,29_ = 4.5, *P* = 0.04). Thus, at current nutrient levels, predicted increases in year-to-year variability of precipitation volume and intensity from climate change—as well as the concordant number of “dry days” (35)—will lead to amplification of boom–bust cycles in widgeongrass cover: alternating between high in dry years (as indicated in recent peaks) and low in wet years.

While light availability limits all seagrass species, widgeongrass meadows are more likely to totally collapse in a poor light environment because of both higher light requirements [i.e., light compensation point of 49.5 μmol m^−2^ s^−1^ compared to 17.95 μmol m^−2^ s^−1^ for eelgrass (36)] and a shorter canopy (5 to 20 cm, vs. 30 to 50 cm eelgrass blade height) that does not reach high into the water column until midsummer (37). Further, widgeongrass has a less extensive root and rhizome system than eelgrass, increasing its susceptibility to mechanical disturbance and increased wave energy (37, 38). Despite these sensitivities, widgeongrass has opportunistic traits like fast shoot turnover, achievement of sexual reproduction within months of germination, and hardy seeds that create a long-lasting seed bank available to fuel rapid postdisturbance recovery (39, 40). Additionally, widgeongrass adaptations to warmer temperatures and longer growing seasons in the modern Chesapeake Bay are likely aiding widgeongrass expansion; widgeongrass has a 7.4 °C higher optimal growing temperature than eelgrass (41, 42), reaches peak biomass during summer when heatwaves affect eelgrass most (37), and outcompetes eelgrass in warm shallow waters (28).

To both conserve multiple seagrass species simultaneously and to keep pace with rapid year-on-year changes in cover and services that result from a more temporally variable dominant seagrass, we call for a parallel shift in coastal monitoring and evaluation of management successes and failures, using the Chesapeake Bay as an example. First, recognizing dominant species identity shifts through long-term monitoring programs requires the adoption of coordinated and standardized on-the-ground surveys to assess changes in composition to complement geographic surveys measuring changes in total extent. More resolved species composition data will allow managers to estimate variation in species-specific stressor responses, particularly in mixed assemblages (a constraint that necessitated our focus on monocultures of these two species), and also identify compositional shifts and invasions as they occur. Species distribution data and projections can help identify where more intense monitoring should focus. For example, recent studies have used climate change and sea level rise projections to predict when and where shifts and expansions may occur in West Africa and the Artic, and such outputs can help to target monitoring efforts (16, 43). To incorporate both spatial distribution and species composition into ecological assessment, the Chesapeake Bay managers are now rolling out a three-tiered, hierarchical monitoring model (44) involving detailed SAV surveys at ~20 permanent Sentinel Sites throughout the bay (Tier 3) and broadly distributed species observations by volunteer scientists (Tier 2) that will supplement the long-standing aerial survey (Tier 1).

As coastal management increasingly recognizes that many seagrass meadows are not monospecific, individual species-specific recovery goals must also be developed across coastlines where multiple seagrass species co-occur and respond to climate differently, particularly in diverse tropical and subtropical assemblages on the brink of change. For the thousands of hectares of the Chesapeake Bay now dominated by widgeongrass, both the current acreage restoration goals based on historic 1930s eelgrass meadow distribution and current water clarity attainment criteria [derived from eelgrass’ light requirements (36)] must be updated for widgeongrass. In seagrass meadows susceptible to boom–bust cycles, water clarity criteria may actually need to be more stringent to support germination and seedling survival, in addition to focusing on persistence of mature beds (45). In our study, wastewater treatment upgrades and stricter power plant emissions standards have effectively reduced point source nutrient pollution and benefited widgeongrass (46), but continuing to reduce nutrient and sediment loads from agricultural fertilizer (e.g., through vegetated buffer installation) and urban runoff (e.g., from improved stormwater management infrastructure) across the watershed will reduce variability in widgeongrass across the region to spur further expansion (47, 48). Our results emphasize the application of best management practices (BMPs) to target these nonpoint sources and urge for an increase in jurisdictional capacity for both BMP implementation and enforcement. Examples of local action that counteract global change can influence both public opinion and policy, while committing to further actions that increase seagrass resistance and recovery rate will support adaptive vegetated coastlines around the world.

In many seagrass meadows, climate change has caused ecosystem-scale shifts to tolerant, opportunistic species that thrive in response to management. In Tampa Bay, large-scale nutrient reductions since the 1970s have facilitated an unprecedented seagrass recovery fueled mainly by expansions in rapidly recolonizing shoal grass (*Halodule wrightii*) and manatee grass (*Syringodium filiforme*), as opposed to the formerly dominant climax species turtle grass (*Thalassia testidinum*) (49). Like the Chesapeake Bay, the new dominants do not appear to be resistant to pulses of nonpoint source nutrient-loaded stormwater, as evidenced by a recent retraction of seagrass below restoration targets for the first time in a decade (50). In the Wadden Sea, the historically dominant subtidal eelgrass never recovered from early twentieth-century die-back but, following management to drastically stabilize sediment and lower eutrophication, intertidal, temperature-tolerant dwarf eelgrass (*Zostera noltii*) meadows persist in the same locations they did nearly a century ago (51–53). Globally, meadows dominated by opportunistic and colonizing seagrasses are both most likely to experience rapid expansion and rapid contraction (54), suggesting that the role of regional water quality management is even more important under climate change to limit the scale of occasional die-backs, prime local meadows for recovery, and maintain important ecosystem functions.

While the full extent of the consequences of shifting dominant seagrass species identities worldwide are not yet quantified, examples from climate change-driven shifts in foundation species identity in other marine and terrestrial ecosystems suggest that functional trade-offs depend on the magnitude and scale of changes to positive interactions, habitat cascades, and food web facilitation. For example, the replacement of cold-water kelp (*Laminaria hyperborea*) by heat-tolerant golden kelp (*Laminaria orchroleuca*) causes orders-of-magnitude losses in structure and function of associated fauna that support a diverse food web (20, 55). Similarly, the temperature-facilitated shift from blue mussels (*Mytilus edulis*) to invasive oysters (*Crassostrea gigas*) in the Wadden Sea changed sediment composition, infauna and epifauna, and dominance of associated crabs and snails enough to have both positive and negative knock-on effects on bird foraging behavior (56, 57). Across forests of eastern North America, climate change and introduced pests have led to the replacement of Eastern hemlock (*Tsuga canadensis*) by a variety of different hardwoods that have drastically altered nitrogen dynamics, and changed associated bird communities in the forests and insect communities in nearby watersheds (17, 58, 59). Overall, replacement by climate change-tolerant, opportunistic species represents both challenges and opportunities for management to conserve ecosystems as we begin to understand the consequences of selecting for certain foundation species life history traits.

Focusing on the Chesapeake Bay, differences in traits and life history strategies between eelgrass and widgeongrass, combined with increased variability in annual extent described above, may jeopardize consistent maintenance of ecosystem functioning. For example, opportunistic seagrasses often have high community metabolism [e.g., widgeongrass metabolism greatly outpaces eelgrass with ~2× median gross primary production and net community production (60)] but a higher above- to below-ground biomass ratio, potentially threatening future blue carbon sequestration (61). Widgeongrass has 50 to 95% lower belowground biomass than eelgrass and thin rod-like shoots that only occupy the water column for 10 to 30% of the year [vs. eelgrass’ thick, strap-like blades in the water column 80% of the year (37)]. These traits contribute to the high annual variability (Fig. 2*C*) that may result in intermittent organic matter resuspension and remineralization over time. Yet, as widgeongrass’ contribution to blue carbon sequestration has not been quantified, we highlight this emerging research need to evaluate whether opportunistic life histories cause a net loss in sediment carbon stocks despite high primary productivity rates.

While the consequences of shifts in seagrass identity in the Chesapeake Bay to food webs are unknown, examples from other regions suggest that the long-term response of fisheries to shifting seagrasses is linked to habitat consistency; after large-scale *Z. marina* die-off in Portugal, temperature-tolerant dwarf eelgrass (*Z. noltii*) is now dominant but supports lower long-term fish diversity and abundance because of interannual cover fluctuations (62). In the Chesapeake Bay, widegongrass’ short leaf height and size require higher shoot densities to provide comparable canopy structure and prey refuge to eelgrass (30, 31), but temporal differences in emergence time [i.e., early spring eelgrass emergence vs. summer widgeongrass emergence (37)] could result in lack of habitat altogether for important transient or juvenile species like blue crabs (*Callinectes sapidus*), spot (*Leiostomus xanthurus*), mysids (*Neomysis spp.*), or Black Sea Bass (*Centropristis striata*) that immigrate into the bay during the spring months. Across latitudes, climate change may even shift trophic pathways as range expansion of herbivorous fish and megafauna are projected to increase top-down control strength into the future (15). Preparing coastal management to be adaptive to novel ecological change thus requires quantifying how seagrass biodiversity, fishery species, juvenile settlement, and blue carbon will respond to climate-caused shifts.

Despite the established value of foundation species for functioning and biodiversity across ecosystems, regulatory agencies without detailed monitoring data often focus on rare species (63) or, when foundation species are protected, may be forced to lump together species with different ecological traits into single “stocks” (e.g., “hectares of seagrass,” “acres of marsh” or just “coastal wetlands”) with little capacity to manage for individual habitat requirements. Our study of two co-occurring and globally distributed seagrasses in a high-priority estuarine habitat emphasizes the importance of adaptively managing for discrete foundation species under climate change. Consequences of species-specific differences in response to climate change, tolerance to local environmental variability, and recovery capacity press habitat conservation to now take advantage of variation among species ecology, ecosystem services, and management requirements to prepare for and adapt to global change.

## Methods

### Documenting Dominant Seagrass Foundation Species Cover Over Time in the Chesapeake Bay.

To describe how the total area of the two major Chesapeake Bay seagrass foundation species, widgeongrass and eelgrass, has changed over time, we first used observations from Virginia Institute of Marine Science’s SAV ground observation dataset (https://www.vims.edu/research/units/programs/sav/access/tables/ground_survey/index.php) to identify and map the individual seagrass meadows throughout the bay that have either remained a widgeongrass monoculture or an eelgrass monoculture throughout our study period. This ground observation dataset consists of presence/absence field surveys of SAV species collected haphazardly throughout the bay by researchers, managers, and citizen scientists since 1978 (22, 64). Widgeongrass can occur in mixed meadows with eelgrass in higher salinity parts of the bay (690 ha of habitat in 2019) and in mixed meadows with freshwater plants in low salinity parts of the bay (883 ha of habitat in 2019) (65), but these areas were excluded from all analyses, and after their removal, we are confident that our analyses focuses specifically on widgeongrass monocultures (9,089 ha in 2019) or eelgrass monocultures (5,299 ha in 2019).

To quantify annual change in density-weighted seagrass meadow cover in our identified widgeongrass and eelgrass monocultures, we analyzed annual aerial imagery from 1984 to 2019, except for 1988, as part of the Virginia Institute of Marine Science SAV Monitoring Program. To determine the annual areal extent and density of each identified widgeongrass bed and eelgrass bed in the Chesapeake Bay, we extracted aerial cover data and summed these meadows to calculate total area (ha) of widgeongrass and eelgrass monoculture each year. To calculate total Chesapeake Bay SAV cover over time (i.e., coverage of all 23 species), we summed the area of all species to quantify total areal cover, data that is publicly available at https://www.vims.edu/research/units/programs/sav/access/charts/baywide/index.php. We used this total SAV area to calculate the proportion of the Chesapeake’s vegetated area that was widgeongrass or eelgrass monoculture each year. We compared differences in the year-to-year variation between total widgeongrass and eelgrass coverage by calculating the deviance from the long-term mean (i.e., 1984 to 2019) for each species every year and applying a two-sample Kolmogorov-Smirnov test.

### Quantifying Annual Change in Widgeongrass Density Weighted Area Over Time in Subestuaries and in the Main Channels.

To describe annual change in widgeongrass cover in the Chesapeake Bay, we computed the total density-weighted bottom cover of widgeongrass beds in two spatially explicit areas, subestuaries, and main channel, that correspond to our environmental and anthropogenic input data (Subestuary Land-use and Load Data, Main Channel Water Quality Data). Widgeongrass bed area and density class were mapped each year from aerial imagery covering each of 35 distinct subestuaries and covering zones around 51 main channel water quality stations spread throughout the Chesapeake Bay (Fig. 1). Each subestuary is a small embayment with its own local watershed whose mouth drains into the main channel of the Chesapeake Bay or one of its major rivers (e.g., Potomac River). These embayments are more tightly linked to their local watershed than tidal influences from the main channel and can be treated as an independent replicates in statistical analyses (66). For the Main Channel analysis, we created each “site” from the 51 water quality stations distributed throughout the main channel and major tributaries that occur within widgeongrass-monoculture. We created the 51 independent spatial zones, one for each site, by dividing the bottom area in 30 × 30 m grid cells according to distance to the nearest monitoring station. Widgeongrass cover for each site was calculated by summing the vegetated cells within the proximate assigned zone. Main channel water quality station zones have also been treated as independent replicates (as in refs. 26 and 32) and correspond with sampling stations for the Chesapeake Bay Program Water Quality Monitoring Database (http://www.chesapeakebay.net/data).

To explicitly understand year-to-year widgeongrass dynamics, to account for variation in the size of sites, and to ensure that large seagrass beds are not overrepresented in data analyses, we calculated the annual proportional change in density-weighted bottom coverage at each site. At each site [i.e., at each of the 35 subestuaries from 1984 to 2015 (n = 649) and 51 stations from 1984 to 2019 (n = 1,041)] every year, we computed total density-weighted bottom cover by multiplying the site area by the midpoint of its percent cover class (very sparse - 5%, sparse – 25%, dense – 55%, very dense – 85%). Then, to compute the proportional change in density-weighted bottom cover, we quantified the total potential habitable area for seagrass at each site by calculating the maximum density-weighted mean composite area over our 35 y of data. Using this maximum value, we scaled widgeongrass coverage at each site each year between 0 and 1, where 1 was the maximum density-weighted mean composite area and 0 was no cover. Finally, we calculated proportional change in scaled density-weighted mean area between two consecutive years at each site.

### Subestuary Analysis: Watershed Inputs and Land-Use in Widgeongrass-Dominated Subestuaries of the Chesapeake Bay.

To understand how watershed inputs and land-use changes affect annual widgeongrass coverage, we first calculated freshwater inputs and associated nutrient and sediment loading variables from 1984 to 2015 within 35 subestuaries dominated by widgeongrass. In each subestuary, we calculated freshwater discharge (“flow,” m^−3^), point and nonpoint source nitrogen and phosphorus loads (kg), and total suspended solid loads (kg) using the Chesapeake Bay Program’s Phase 6 watershed model (http://www.chesapeakebay.net/groups/group/modeling_team). These water discharge and load variables were simulated daily from 1984 to 2015 at a subcounty scale and were summed for each subestuary in each growing season (April to August). Next, we calculated annual land-use variables, expressed as the proportion of the subestuary catchment utilized for agriculture (i.e., areas of crop, pasture, hay, and grazing lands) or development (i.e., commercial, industrial, recreational, and residential lands) each year, using the United States Geological Survey National Water Quality Assessment Wall-to-Wall Anthropogenic Land Use Trends dataset (https://pubs.er.usgs.gov/publication/ds948) (67). We then calculated total annual growing season loads for each watershed and land-use variable to use in our subestuary structural equation analysis.

### Main Channel Analysis: Water Column Biogeochemistry in Widgeongrass-Dominated Sites in the Main Channel Chesapeake Bay.

To determine how water quality affects annual widgeongrass coverage in the major rivers and channels of the Chesapeake Bay, we summarized seven variables collected bi-weekly at the 51 water quality monitoring stations from 1984 to 2019. At each station, we calculated springtime (March to May) mean values for each of the following variables: temperature, salinity, turbidity (Secchi depth), and water column concentrations of total nitrogen and total phosphorus, and chlorophyll-*a*. While measurements are taken at multiple depths, we used values at 0.5 or 1 m depth to best reflect conditions in nearshore shallow waters. At each station, temperature and salinity were measured along a hydrographic profile with a multiparameter sonde. Water clarity was measured by lowering a black and white Secchi disk over the side of the sampling vessel with a measuring line and recorded the depth at which the observer could no longer distinguish between the black and white zones of the disc. Water samples were collected from the surface and several depths and sent to a laboratory for analysis of nutrient concentrations, chlorophyll-a, and total suspected solids. For the main channel analysis, we calculated mean, median, maximum, minimum, range, and step-wise change values for each variable and summarized each variable into seasons (i.e., annual, spring, summer, fall, winter). We found that springtime means were the best predictors of widgeongrass change.

### Structural Equation Modeling: Effect of Watershed Inputs and Land-Use (Subestuary Analysis), and the Effect of Water Column Biogeochemistry (Main Channel Analysis) on Widgeongrass Change in the Chesapeake Bay.

We quantified how rapidly changing environmental and anthropogenic factors in the watershed and water column control annual widgeongrass meadow coverage in the Chesapeake Bay by fitting structural equation models to describe: 1) the effect of long-term indicators of land use and nutrient loadings from watershed models on annual change in areal coverage in each of the 35 widgeongrass-dominated subestuaries and embayments (Subestuary Analysis; ~8,000 total ha), and 2) the effect of seasonal water quality on annual change in areal coverage in each of the 51 monitoring stations in major rivers and channels (Main channel Analysis; ~18,000 total ha). Structural equation modeling (SEM) allows for fitting complex networks where variables can be both predictors and responses, facilitating the identification of cascading effects. First, we determined the effect of watershed inputs and land-use on inter-annual change in widgeongrass area in Chesapeake Bay subestuaries. For this subestuary SEM, we identified plausible causal links between land-use characteristics and estimated loads based on knowledge of the system and expert opinion (*SI Appendix*, Fig. S1). Next for the main channel SEM, we analyzed the effect of water quality on widgeongrass change with a different conceptual structure (*SI Appendix*, Fig. S2) due to a different set of environmental variables, also derived from a priori knowledge of the system.

Because of the known importance of previous year’s seagrass density on the current year’s seagrass density, we explicitly considered the interaction between all variables and the recorded density in the previous year at each site. For example, in the main channel analysis, we tested both the direct effect of chlorophyll-*a* on widgeongrass change (widgeongrass change ~ chl-*a*) and its interactive effect with previous year’s proportional grass coverage (widgeongrass change ~ chl-*a* * widgeongrass density _y−1_). Additionally, because of the boom/bust nature of widgeongrass and due to the spatial configuration of our monitoring stations, many vegetated sites also contained no grass for several years or have only ever contained a very small amount of grass. To reduce the effect of zeroes on our models, we filtered out data points where no grass was recorded for three years in a row. This data filtering left us with 491 observations from the Subestuaries (1984 to 2015) and 1,041 observations from the Main channel (1984 to 2019, excluding 1,988). We tested our structural equation models by using datasets with no zero-filtering and also with all zeros filtered out and found only minimal differences in model fit, with only changes in effect size and not model significance.

Because seagrass cover is temporally and spatially autocorrelated, we modeled cover using linear mixed effects models in a piecewise SEM, using the piecewiseSEM package (68), a variant of SEM that uses local estimation and can incorporate statistical interactions and mixed effects. Models were fit with subestuary or station (i.e., site) as a random effect, and an additional site autoregressive moving average correlation structure. Plotting the correlation among residuals as a function of the time lag revealed that the random effect and correlation structure eliminated any autocorrelation among the data points, such that the data points could be treated as truly independent replicates. Model assumptions were assessed visually, and we calculated individual model R^2^ values (R^2^marginal is the fixed effect only statistic, R^2^conditional is the full model statistic including both fixed and random effects). We assessed global goodness-of-fit using Fisher’s *C* and computed standardized path coefficients, which are scaled by the SDs of the variables involved. Thus, the standardized coefficients are unitless measures of association that can be compared across different relationships within and among models. Unstandardized coefficients included in the *SI Appendix* can be used to compare magnitude of effects across different populations irrespective of variance. All analyses were conducted in the R statistical software version 4.1.0 (69), and all code and data used in these above and below analyses are available in a public repository (https://github.com/mhensel/Ruppia-Change).

### Main Channel Freshwater Discharge Data: Correlations Between Susquehanna River Flow and Annual Widgeongrass Cover.

While flow from each subestuary was included in our subestuary analysis and salinity is a strong indicator of site-by-site freshwater input in our main channel analysis, we also examined how variation in flow from the largest source of freshwater flow into the main channel Bay (i.e., the Susquehanna river) correlates with interannual variation in widgeongrass cover across the Bay. To evaluate this relationship, we accessed daily discharge data from the Connowingo Dam (https://waterdata.usgs.gov/md/nwis/uv?site_no=01578310) and calculated mean annual and seasonal discharge, for the spring (March to May) and winter (December to February) of each year from 1984 to 2019. We analyzed the relationship between logged Susquehanna River annual, wintertime, and springtime discharge (cubic feet per second per day) and annual areal change in total widgeongrass cover across the Bay using a linear model. We evaluated assumptions using the performance package in R (70) and found no violations.

## Supplementary Material

Appendix 01 (PDF)

## Data Availability

Environmental data, aerial survey data and all code for all analyses and figures data have been deposited in https://github.com/mhensel/Ruppia-Change (71).
