# Supplementary material for: Rise of Ruppia in Chesapeake Bay: Climate change–driven turnover of foundation species creates new threats and management opportunities
Source: Proc Natl Acad Sci U S A. 2023 May 30;120(23):e2220678120. doi: 10.1073/pnas.2220678120 (PMC10265998; doi:10.1073/pnas.2220678120)
Supplement: Supplementary file 1 — Appendix 01 (PDF) [file pnas.2220678120.sapp.pdf]

Supporting Information

VIMS Submersed Aquatic Vegetation Monitoring Program

Submersed Aquatic Vegetation (SAV) bed area and density class were mapped from aerial imagery acquired annually from 1984 through 2019 (except for 1988) as part of the Virginia Institute of Marine Science Submersed Aquatic Vegetation Monitoring Program (https://www.vims.edu/sav). Panchromatic photography at a scale of 1:24,000; 60% flightline overlap and 20% sidelap was acquired with a standard mapping camera until 2014. From 2014-2015, multispectral imagery was acquired using a digital mapping camera with a ground sample distance of 24 cm. Acquisition conditions (including tidal stage, plant growth, sun angle, atmospheric transparency, water turbidity, and wind) were selected to optimize the visibility of SAV beds. For our widgeongrass and eelgrass monoculture zones, acquisition generally occurred from June-August.

SAV beds were originally hand-traced SAV bed outlines directly from photographs, mapped onto a translucent United States Geological Survey 7.5-minute quadrangle maps. Bed boundaries were digitized into a geographic information system (GIS) dataset. In 2001, the aerial photography negatives began to be scanned and in 2014 a digital sensor was introduced eliminating film altogether. The scanned and new digital imagery is orthorectified using ERDAS LPS image-processing software (ERDAS, Atlanta GA). SAV bed boundaries are photo-interpreted directly on-screen, maintaining a fixed scale using ESRI ArcMap GIS software (ESRI, Redlands CA). The spatial accuracy of the dataset varies from approximately ± 24 m (earlier data) to approximately ± 4 m (recent data).

Subestuary Analyses Variables: calculating loads for each subestuary

Freshwater discharge, point and non-point source nitrogen, phosphorus, and total suspended solid loads were estimated using the Chesapeake Bay Program's Phase 6 beta 4 watershed model (CBWM) (http://www.chesapeakebay.net/groups/group/modeling_team). The CBWM is an open-source model developed by multiple state, federal, and academic institutions along with other stakeholders within the Chesapeake Bay Program partnership. The CBWM incorporates information on land use, fertilizer applications, wastewater plant discharges, septic systems, atmospheric deposition, farm animal populations, weather, hydrology, and best management practices into multiple sub-models to determine the total loads of nutrient and sediments reaching the Chesapeake Bay across all terrestrial, atmospheric, and direct inputs.

In each subestuary, we calculate an estimated annual concentration (C) of each nutrient load as

$$C = \frac{fm}{f + p}$$

where, *f* is flow rate (volume/day), *m* is the material input concentration, and *p* is the tidal exchange rate (volume/day). We used this to assemble the total growing season (April-August) discharge of each material from the watershed of each subestuary.

Main channel Analyses Variables: calculating water quality variables

The main channel analysis determines how local water quality impacts widgeongrass cover throughout the widgeongrass-dominated areas in the Chesapeake Bay by examining *in situ* water quality data from nearby sampling stations in the Chesapeake Bay Program Water Quality Monitoring Database (http://www.chesapeakebay.net/data). Known methodological changes over the analysis period for TP and TN required additional corrections published in Lefcheck et al SI (1).

Structural Equation Modeling

To reflect conditions in the subestuaries and main bay related to the date of observation for SAV, we examined annual, spring, summer, and growing season environmental data and found that mean-summarized springtime environmental data (March-May) best explained widgeongrass change. For the subestuary analysis, growing season (April-August) loads were calculated to estimate the amounts of nutrients and sediment entering the watershed and the subestuary. For the main bay analysis, water quality variables were averaged over those periods. Any site and year combination for which any variable was not recorded was discarded, and any set of three years with no SAV found was discarded, leaving a total of N = 649 observations for the subestuary analysis over 30 years (1985-2015, omitting 1988), and N = 1041 observations for the main bay analysis over 34 years (1985-2019, omitting 1988).

We used structural equation modeling (SEM) to analyze both the direct and indirect controls on widgeongrass in two different causal networks (Figs. S2, S3). SEM is a powerful tool for understanding cascading effects, such as from the watershed to shallow water seagrass habitat, because variables can be both predictors and responses. Piecewise SEM, or local estimation, allows variables to be modeled to a wide variety of distributions and hierarchical structures, and incorporates statistical interactions better than in previous iterations of SEM. A key feature of SEM is that it assumes causal (i.e., directional) relationships. One can support causal inferences in several ways, including previous experimental and observational evidence, biological knowledge, and logical intuition about the system. Given how well the Chesapeake Bay has been monitored and studied, we have a higher degree of confidence in the causal nature of the associations identified in our data. Such expert knowledge is an inherent feature in the structuring and evaluation of multivariate causal hypotheses. In addition, we have carefully structured our models to improve causal inference. The ‘back-door criterion’ proposes that adding covariates that explicitly block the confounding effect of other factors on the response of interest can be used to open the possibility of causal linkages. For example, nutrients can affect widgeongrass change directly or indirectly by increasing chl-*a* or decreasing water clarity. In other words, these linkages to nutrients provide a statistical control that reduces the probability of spurious correlation.

For the subestuary SEM, we identified plausible causal links between land use characteristics, fertilizer and manure application, and estimated loads based on knowledge of the system and expert opinion (Fig. S2). For the main bay, we tested a different conceptual structure (Fig. S3) but again used generalized linear mixed effects models with ARMA correlation structures for the individual models. As with the subestuary analyses, the relationships specified in the main bay analysis were derived from *a priori* knowledge of widgeongrass systems. Both SEMs generated a set of meaningful independence claims, so we could assess global goodness-of-fit using Fisher's *C*.

For both sets of SEMs, we computed standardized path coefficients. These are scaled by the standard deviations of the variables involved, so the standardized coefficients are unitless measures of association that can be compared across the same relationship in different models and across different relationships within and among models. Standardized coefficients are also useful for computing indirect effects. Because they are unitless, the strength of indirect pathways can be obtained by multiplying the standardized coefficients along the path. For example, we can compute the indirect pathway from phosphorus to chlorophyll-*a* to water clarity to widgeongrass by multiplying the path coefficients.

Supporting Tables

Table S1: Trait table, summarizing several plant traits for widgeongrass (*Ruppia maritima)* and eelgrass (*Zostera marina*) in Chesapeake Bay

**Trait**	**Widgeongrass**	**Eelgrass**	**Widge source**	**Eelgrass source**
Temperature, Maximum	>30 °C	28 °C	(1)	(2)
Temperature, Optimal	30 °C	22.6 °C	(3)	(4)
Light compensation point (Ic, μmol m^{-2} s^{-1})	11 to 88	0.9 - 35	(5)	(5)
Irradiance saturation point (Ik μmol/ $m^{-2}s^{-1}$)	45-1200	7-700	(5)	(5)
Q10 temperature coefficient	1.6	1.52	(1)	(6)
Seed bank length	Years to decades	Days to weeks	(7)	(7)
Plant height, peak growing season mean (cm)	45	50	(5)	(5)
Mean shoot emergence time	March-April	June-July	(8)	(8)
Leaf area, mean (mm^2)	44.176	3162	(9)	(9)
Leaf dry mass (mg)	0.79	10.712	(9)	(9)
Leaf dry matter content (LDMC, mg/mm^2)	0.21	0.12	(9)	(9)
Maximum **|** Mean **|** Minimum salinity observed for taxa	25.75 **|** 13.36 **|** 1.68	25.75 **|** 17.58 **|** 9.62	(10)	(10)
Percent fines sediment/sediment type	mud or sand	8.1-28.8	(5)	(5)
Percent organics in sediment	2 - 3.25%	1.25 - 5.8 %	(5)	(5)
Pollen diameter, mean (μm)	70	2550	(9)	(9)

1. A. S. Evans, K. L. Webb, P. A. Penhale, Photosynthetic temperature acclimation in two coexisting seagrasses, Zostera marina L. and Ruppia maritima L. *Aquatic Botany* **24**, 185–197 (1986).
2. E. C. Shields, D. Parrish, K. Moore, Short-Term Temperature Stress Results in Seagrass Community Shift in a Temperate Estuary. *Estuaries and Coasts* **42**, 755–764 (2019).
3. C. Cerco, K. A. Moore, System-Wide Submerged Aquatic Vegetation Model for Chesapeake Bay. *Estuaries* **24**, 522–534 (2001).
4. C. Leuschner, U. Rees, CO2 gas exchange of two intertidal seagrass species, Zostera marina L. and Zostera notii Hornem., during emersion. *Aquatic Botany* **45**, 53–62 (1993).

5. R. A. Batiuk, *et al.*, “Chesapeake Bay Submerged Aquatic Vegetation Water Quality and Habitat-Based Requirementsand Restoration Targets: A Second Technical Synthesis” (Chesapeake Bay Program, 2000).
6. J. A. Marsh, W. C. Dennison, R. S. Alberte, Effects of temperature on photosynthesis and respiration in eelgrass (Zostera marina L.). *Journal of Experimental Marine Biology and Ecology* **101**, 257–267 (1986).
7. K. Kilminster, *et al.*, Unravelling complexity in seagrass systems for management: Australia as a microcosm. *Science of The Total Environment* **534**, 97–109 (2015).
8. K. A. Moore, Influence of Seagrasses on Water Quality in Shallow Regions of the Lower Chesapeake Bay. *Journal of Coastal Research* **10045**, 162–178 (2004).
9. J. Kattge, *et al.*, TRY plant trait database – enhanced coverage and open access. *Global Change Biology* **36**, 119–188 (2020).
10. C. J. Patrick, D. E. Weller, R. J. Orth, D. J. Wilcox, M. P. Hannam, Land Use and Salinity Drive Changes in SAV Abundance and Community Composition. *Estuaries and Coasts* **41**, 85–100 (2017).

Table S2: Regression coefficient table for subestuary structural equation model. Widgeongrass change is the proportional change in widgeongrass area per site and Widgeongrass density$_{y-1}$ is the scaled widgeongrass coverage in that site in the previous year. All values are springtime (March-May) means and have been log$_{10}$-transformed. Asterisk (*) indicates interaction term, i.e., Widgeongrass$_{y-1}$ * Flow is the interaction between the previous year's grass coverage and water flow rates. Abbreviations in this table include: Total Suspended Solids (TSS), non-point source nitrogen and phosphorus (NPTN, NTPT), point source total nitrogen and total phosphorus (PTN, PTP). Standardized coefficients (scaled by standard deviations) of significant values ($P < 0.05$) are ***bolded and italicized***. Correlations included in the model are at the bottom of the table and are *italicized.*.

Response	*Predictor*	*Estimate*	*Std.Error*	*DF*	*Crit.Value*	*P.Value*	*Std.Estimate*
TSS	***Flow***	***2.31***	***0.0468***	***435***	***49.3***	***0***	***0.948***
TSS	% Agriculture	0.0047	0.0086	435	0.549	0.583	0.052
TSS	***% Developed***	***0.0089***	***0.0035***	***435***	***2.55***	***0.0111***	***0.144***
NPTN	***Flow***	***1.8***	***0.0088***	***435***	***205***	***0***	***1.11***
NPTN	***% Agriculture***	***0.0103***	***0.0045***	***435***	***2.27***	***0.0234***	***0.172***
NPTN	% Developed	0	0.001	435	0.0124	0.99	0.0003
PTP	***Flow***	***1.29***	***0.058***	***435***	***22.2***	***0***	***0.635***
PTP	% Developed	-0.0005	0.0047	435	-0.0966	0.923	-0.0089
PTP	% Agriculture	0.0142	0.0105	435	1.35	0.177	0.189
Δ Widgeongrass	***Widgeongrass density y-1***	***-2.45***	***0.6277***	***429***	***-3.9***	***0.0001***	***-2.33***
Δ Widgeongrass	Flow	-0.0881	0.0641	429	-1.37	0.17	-0.26
Δ Widgeongrass	NPTN	0.0054	0.0481	429	0.112	0.911	0.0259
Δ Widgeongrass	TSS	0.0224	0.0246	429	0.911	0.363	0.162
Δ Widgeongrass	PTP	0.0105	0.0164	429	0.638	0.524	0.0627
Δ Widgeongrass	***Widgeongrassy -1 * PTP***	***-0.111***	***0.0583***	***429***	***-1.9***	***0.0575***	***-0.106***
Δ Widgeongrass	Widgeongrassy-1 * Flow	0.412	0.2471	429	1.67	0.0964	1.22
Δ Widgeongrass	***Widgeongrassy -1 * NPTN***	***-0.412***	***0.1794***	***429***	***-2.3***	***0.0222***	***-1.97***

Δ Widgeongrass	Widgeongrassy-1 * TSS	0.151	0.0882	429	1.71	0.0883	1.09
NPTN	*TSS*	*0.306*	*-*	*472*	*6.97*	*0*	*0.306*

Table S3: Coefficients of determination (R^2) for subestuary structural equation model of watershed inputs on widgeongrass change in subestuaries and embayments. Marginal R^2 reports only the variance explained by fixed effect; conditional R^2 reports the variance explained by both fixed and random effects.

Response	*Marginal R^2*	*Conditional R^2*
Total suspended solids	0.75	0.96
Non-point source nitrogen	0.8	0.99
Point source phosphorus	0.39	0.83
Δ Widgeongrass	0.46	0.49

Table S4: Regression coefficient table for main bay structural equation model. Widgeongrass change is the proportional change in widgeongrass area per site and Widgeongrass density$_{y-1}$ is the scaled widgeongrass coverage in that site in the previous year. All values are springtime (March-May) means and have been log$_{10}$-transformed. Asterisk (*) indicates interaction term, i.e., Widgeongrass$_{y-1}$ * Salinity is the interaction between the previous year's grass coverage and springtime salinity. Abbreviations in this table include: Total Suspended Solids (TSS), chlorophyll-a (chl-A), total nitrogen and total phosphorus (TN, TP), while Turbidity is estimated via Secchi depth. Standardized coefficients (scaled by standard deviations) of significant values (P < 0.05) are ***bolded and italicized***. Correlations included in the model are at the bottom of the table and are *italicized.*

Response	*Predictor*	*Estimate*	*Std.Error*	*DF*	*Crit.Value*	*P.Value*	*Std.Estimate*
chl-A	***Temperature***	***-0.7695***	***0.1329***	***987***	***-5.7911***	***0***	***-0.167***
chl-A	***TP***	***0.678***	***0.0609***	***987***	***11.1351***	***0***	***0.4767***
chl-A	***TN***	***0.2095***	***0.0617***	***987***	***3.3939***	***0.0007***	***0.1554***
Water clarity	***chl-A***	***-0.2149***	***0.0131***	***986***	***-16.3851***	***0***	***-0.3129***
Water clarity	***Temperature***	***-0.1295***	***0.0568***	***986***	***-2.2784***	***0.0229***	***-0.0409***
Water clarity	***TN***	***-0.103***	***0.0308***	***986***	***-3.3441***	***0.0009***	***-0.1112***
Water clarity	***TP***	***-0.2538***	***0.0312***	***986***	***-8.136***	***0***	***-0.2599***
Δ Widgeongrass	***Widgeongrass density y-1***	***-1.1996***	***0.5888***	***977***	***-2.0374***	***0.0419***	***-1.3919***
Δ Widgeongrass	Salinity	-0.0539	0.0462	977	-1.1664	0.2437	-0.0644
Δ Widgeongrass	chl-*A*	-0.0072	0.0241	977	-0.2971	0.7665	-0.0125
Δ Widgeongrass	TP	-0.0228	0.0472	977	-0.4826	0.6295	-0.0279
Δ Widgeongrass	***TN***	***-0.1752***	***0.0488***	***977***	***-3.5906***	***0.0003***	***-0.2267***
Δ Widgeongrass	*Water clarity*	-0.0072	0.0488	977	-0.1469	0.8833	-0.0086
Δ Widgeongrass	Temperature	-0.1065	0.1016	977	-1.0478	0.295	-0.0403
Δ Widgeongrass	Widgeongrass y-1 * Temperature	0.045	0.3886	977	0.1157	0.9079	0.0522
Δ Widgeongrass	***Widgeongrassy-1 * Salinity***	***0.7715***	***0.1967***	***977***	***3.9212***	***0.0001***	***0.9215***
Δ Widgeongrass	***Widgeongrassy-1 * chl-A***	***-0.4847***	***0.092***	***977***	***-5.2672***	***0***	***-0.8457***

Δ Widgeongrass	Widgeongrass y-1 * TP	-0.1959	0.1741	977	-1.1255	0.2607	-0.2404
Δ Widgeongrass	Widgeongrass y-1 * TN	0.1763	0.1938	977	0.9095	0.3633	0.2282
Δ Widgeongrass	Widgeongrass y-1 * *Water clarity*	-0.1838	0.18	977	-1.0209	0.3076	-0.2202
TN	*TP*	*0.5969*	-	*1039*	*23.9835*	*0*	*0.5969*
TN	*Salinity*	*0.1549*	-	*1041*	*5.0507*	*0*	*0.1549*
chl-A	*Salinity*	*-0.1552*	-	*1041*	*-5.0613*	*0*	*-0.1552*

152

Table S5: Coefficients of determination (R^2) values from the main bay structural equation model explaining the effect of springtime water quality on widgeongrass change. Marginal R^2 reports only the variance explained by fixed effects and conditional R^2 reports the variance explained by the fixed and random effects.

Response	*Marginal R^2*	*Conditional R^2*
chl-*a*	0.33	0.40
Water clarity	0.37	0.70
**Δ Widgeongrass**	0.46	0.54

Table S6: Analysis of variance (Type II ANOVA) results from linear model examining the correlation between logged annual, spring, and winter water flow from the Susquehanna River, the major freshwater flow source for the main bay sites, and mean annual widgeongrass change in our main bay sites. Bolded indicates significantly significant p value (alpha = 0.05)

Response: Change in Baywide widgeongrass area	*SumSq*	*df*	*F value*	*p-value*
Spring flow	4910511	1	0.8967	0.3515
Winter flow	3987855	1	0.7282	0.40046
**Annual flow**	**24624447**	**1**	**4.4965**	**0.04264**
Residuals	158815524	29		

Supporting Figures

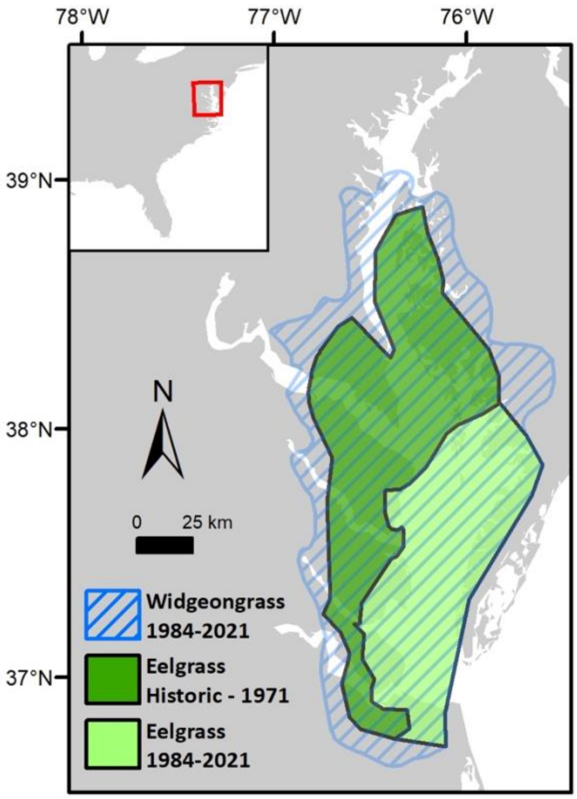

Figure S1: Map of widgeongrass (*Ruppia maritima)* and eelgrass (*Zostera marina*) coverage in the Chesapeake Bay. Historically, eelgrass dominated most of the poly- and meso-haline salinity areas of the Chesapeake Bay (light green and dark green). Eelgrass currently dominates a smaller area in the lower Chesapeake (dark green). Widgeongrass has been observed in varying densities throughout these areas in the Bay from 1984-2021 (blue hashed), but now forms dominant monocultures in the historic eelgrass habitat (dark green and blue hashed). Widgeongrass has become the dominant space-holding plant in the Chesapeake Bay. Note that the middle of the Chesapeake Bay is shaded but is too deep to support seagrass growth, while some species zones overlap land for visual aid.

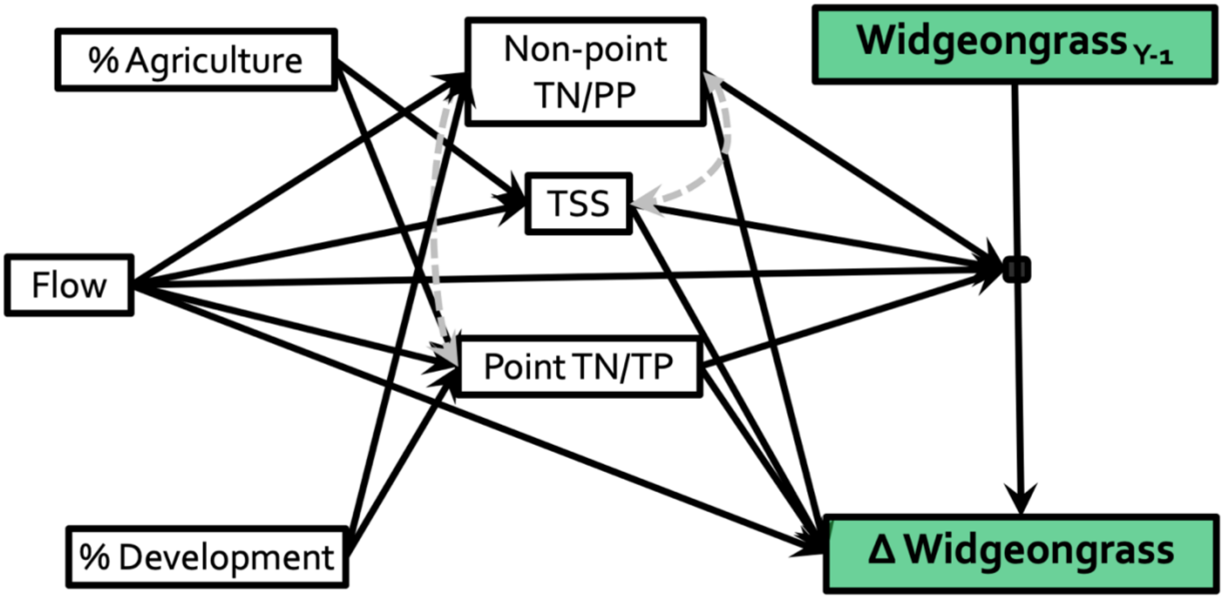

Figure S2: Conceptual structural equation model for the subestuary analysis. Widgeongrass change is predicted by three sets of variables: widgeongrass proportional density in the previous year (Widgeongrass$_{y-1}$ -> Widgeongrass change), directly by environmental variables, and the interaction between environmental variables and widgeongrass density the previous year (e.g., Widgeongrass$_{y-1}$:Non-point source total phosphorus)

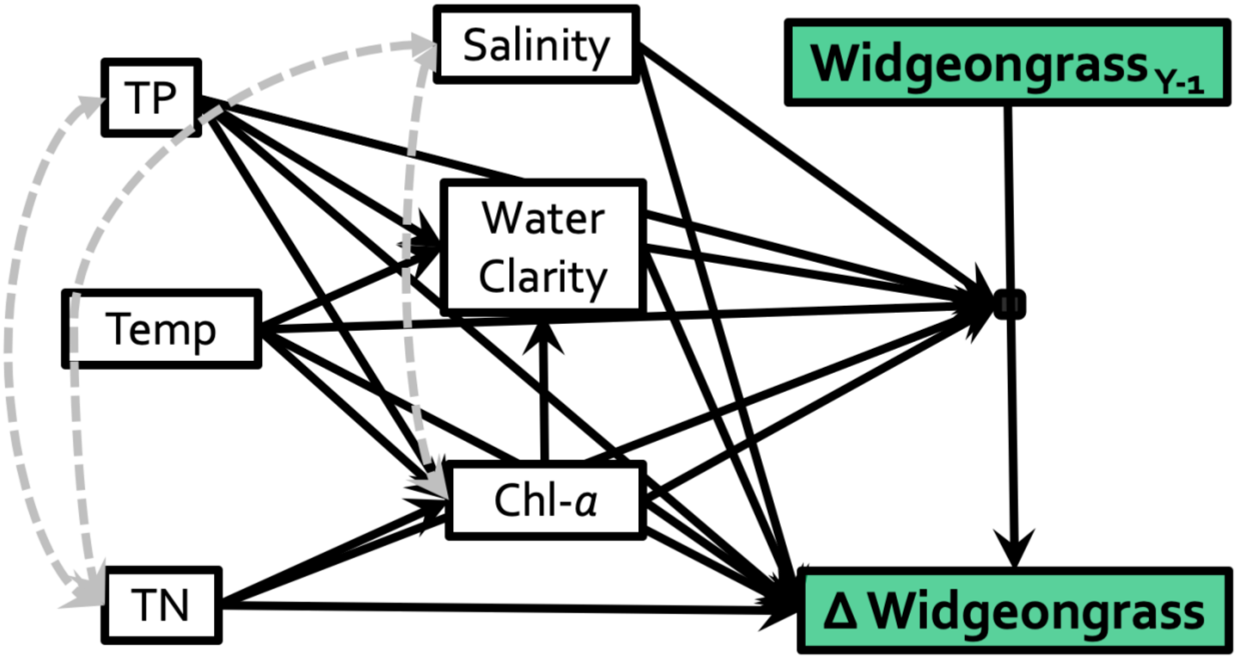

Figure S3: Conceptual SEM for main bay analyses. Widgeongrass change is predicted by three sets of variables: widgeongrass proportional density in the previous year ($Widgeongrass_{y-1}$ -> Widgeongrass change), directly by environmental variables, and the interaction between environmental variables and widgeongrass density the previous year (e.g., $Widgeongrass_{y-1}$:chl-*a*).

SI References

1. J. S. Lefcheck, *et al.*, Long-term nutrient reductions lead to the unprecedented recovery of a temperate coastal region. *Proc Natl Acad Sci USA* **115**, 3658–3662 (2018).
